# Diagnostic Performance of Automated Blood Pressure Monitor for Detection of Atrial Fibrillation

**DOI:** 10.7759/cureus.53093

**Published:** 2024-01-28

**Authors:** Rasid Othman, Ayman Suliman, Zurkurnai Yusof, W Yus Haniff W Isa

**Affiliations:** 1 Internal Medicine, Hospital Universiti Sains Malaysia, Kubang Kerian, MYS; 2 Cardiology Unit, Hospital Universiti Sains Malaysia, Kubang Kerian, MYS; 3 Cardiolgy Unit, Hospital Universiti Sains Malaysia, Kubang Kerian, MYS

**Keywords:** rossmax x5, heart diseases, electrocardiogram, blood pressure monitor, atrial fibrillation

## Abstract

Background

Atrial fibrillation (AF) is a type of heart disease characterized by an irregular cardiac rhythm. The complications of AF are associated with significant morbidity, mortality, and medical expenses. This emphasizes the significance of detecting AF early using a feasible device.

Methods

A total of 123 patients who attended cardiology and INR clinics were enrolled, with 51 of them having AF. The blood pressure of all patients was measured three times using the Rossmax X5, while a single-lead electrocardiogram (ECG) was monitored simultaneously. Following that, a 12-lead ECG was performed on all patients. A cardiologist confirmed the irregular rhythm.

Results

Compared to the 12-lead ECG method, Rossmax X5 has an accuracy of 99.3%, a sensitivity of 100%, and a specificity of 98.6%. The positive and negative predictive values were also significant, which were 98.1% and 100%, respectively.

Conclusion

The Rossmax X5 automated blood pressure monitor has a high detection accuracy for AF. Therefore, Rossmax X5 can be recommended for use in the clinical setting as a screening tool for early AF detection.

## Introduction

Atrial fibrillation (AF) is the most commonly sustained cardiac arrhythmia worldwide [1]. Cerebrovascular accident and congestive heart failure (HF) are commonly associated with AF. Being the most frequent heart rhythm disorder encountered worldwide, the incidence of AF is expected to rise in the future [2]. Besides, it is associated with an increased risk of mortality and morbidity contributed by cerebrovascular accident, CHF, and impaired quality of life. These unwanted outcomes lead to negative socioeconomic and healthcare impacts [3,4].

The most crucial morbidity contributed by AF is stroke. According to the US Preventive Services Task Force (USPSTF), AF is a significant risk factor for ischemic stroke, which can cause a five-fold increased risk for stroke. Around 20% of patients who have a stroke associated with AF are newly diagnosed with AF upon presentation [5]. Therefore, early detection of AF can lead to early assessment and starting appropriate treatment such as anticoagulant prophylaxis in those high-risk patients. It is mentioned that the stroke caused by AF tends to be more severe than that due to other causes [6].

AF is recognized based on the presentation, whether it is acute or chronic, and based on the duration of the episode, whether it is paroxysmal, persistent, long-standing persistent, or permanent [7]. Paroxysmal AF is best detected by using Holter, which is a 24-hour ambulatory ECG because the rhythm is non-sustained in nature and may be missed on 12-lead ECG recording [8].

The common risk factors of AF include being elderly, and certain cardiac diseases such as valvular heart disease, hypertensive heart disease, and others. Most of these patients are asymptomatic for AF [9]. This leads to the idea of creating a suitable method for AF screening, especially in the general population with higher AF risk to detect and treat them early. The current recommended way by the European Society of Cardiology for AF detection is by pulse palpation and ECG rhythm strip [10]. Nevertheless, for the past few years, there has been an emerging usage of blood pressure monitors (BPMs) to detect AF. This unique BPM is integrated with an algorithm for AF detection so that it can be used as a screening tool for those patients with risk factors [10].

Recent guidance from the National Institute for Health and Care Excellence (NICE) advocates the use of automated oscillometric BPMs for the detection of suspected AF in patients being screened or monitored for hypertension. This particular BPM is integrated with an algorithm for AF detection so that it can be used as a screening tool for those patients with risk factors [10]. Automated oscillometric BP monitors may provide accurate information on the irregularity of the cardiac rhythm by measuring the width and amplitude of pulse waves during BP measurement [11].

To date, there are few automated oscillometric BPMs for AF detection in the market. Most of them have around 96-97% sensitivity and >90% specificity to detect AF [12]. Rossmax X5 is one of the automated oscillometric BPMs that is integrated with this function. In Malaysia, there were no reported studies using this device for this purpose. We aim to evaluate the diagnostic value of the Rossmax X5 automated blood pressure monitor (ABPM) as a screening tool for early detection of AF.

This study was submitted to the Universiti Sains Malaysia repository website as a part of a final-year thesis for the Master's degree.

## Materials and methods

This is a cross-sectional study that used an ABPM device (Rossmax X5) to detect its accuracy for AF detection. The study was done from 1st June until 31 July 2020. All participants were recruited from patients who attended cardiology and INR clinics. Patients were selected by convenience sampling method based on the listed inclusion and exclusion criteria.

The inclusion criteria included all patients who received treatment in cardiology and INR clinics, and aged 50 years and above. The exclusion criteria included patients with arm circumference beyond the maximum provided cuff size of 40 cm or more and those with bilateral fistulas or any abnormality of upper limbs that can interfere with BP measurement. Written consent was obtained prior to procedures. Blood pressure measurements were performed based on the recommendations of the American Heart Association, 2005 [13]. The sample size was calculated for each objective by using an online calculator [14].

The participants were sitting on the chair or lying in a supine position on the examination couch during the blood pressure measurement. A blood pressure cuff was applied neatly on the arm, 2 cm above the brachial artery and aligning the "artery mark." Concurrent single-lead ECG monitoring was used during the blood pressure measurement to identify any discrepancy in rhythm detection. The participants were rested for at least 5 minutes before blood pressure measurement. The measurements were done thrice for at least 5 minutes each, and the results were documented. Any abnormal rhythm other than sinus rhythm was shown on the monitor as arrhythmias - atrial fibrillation (ARR-Afib), arrhythmias - premature contraction (ARR-PC), or both. A subsequent confirmatory 12-lead ECG was performed and analyzed. Any abnormal rhythm was further interpreted by the cardiologist.

This study was conducted in accordance with the principles of the Declaration of Helsinki. The patients’ personal identification and clinical data were confidential and were reported as collective information and not on an individual basis. Approval from the Universiti Sains Malaysia Ethics Committee and the National Malaysia Research Registry was obtained prior to the commencement of the study.

The statistical analysis was performed using SPSS software (version 26.0; IBM Corp, Armonk, NY) to check the significance of ABPM in relation to ECG. The numerical data was represented in numbers (mean ± standard deviation). The correlation of ABPM vs ECG was tested using the chi-square test. A p-value of <0.05 was considered statistically significant.

## Results

Demographic data

A total of 123 participants were recruited in this study, with male participants constituting 71.5% of all participants. The mean age was 65.07 (1.53) years. Among all subjects, 41.46% have AF. The most common comorbidity in our participants was hypertension, followed by ischemic heart disease, dyslipidemia, diabetes mellitus, and other diseases (Table 1).

**Table 1:** Background characteristics of study participants Data presented as mean (SD). *Data presented as number (n).

Parameters	All participants (N = 123)	Participants with AF (n = 51)	Participants without AF (n = 72)
Age (years)	65.07 (1.53)	66.33 (2.35)	64.18 (2.06)
Gender (male/female)	88/35	27/24	61/11
Systolic blood pressure (mmHg)		135 (5.9)	138 (4.4)
Diastolic blood pressure (mmHg)		78 (3.3)	78 (1.9)
Comorbidity^*^			
Hypertension	87	28	59
Ischemic heart disease	80	25	55
Dyslipidemia	50	14	36
Type 2 diabetes mellitus	37	8	29
Stroke	13	7	6
Chronic kidney disease	13	5	8
Chronic rheumatic heart disease	12	7	5
Pulmonary disease (chronic obstructive pulmonary disease, bronchial asthma)	5	3	2
Hyperthyroidism	4	4	0
Cardiomyopathy	2	1	1

Detection of AF by Rossmax X5

The accuracy index of the Rossmax X5 ABPM for AF detection was about 99%. The sensitivity and specificity were 100% and 98.6%, respectively (Table 2). Out of 123 patients recruited, only one false-positive result was detected while the positive and negative predictive values were 98.1% and 100%, respectively (Table 3).

**Table 2:** Result of the accuracy of Rossmax X5 in detecting atrial fibrillation

	Disease				
Test	Present	N	Absent	n	Total
Positive	True positive	51	False positive	1	52
Negative	False negative	0	True negative	71	71
Total	True positive + false negative	51	False positive + true negative	72	123

**Table 3:** Sensitivity and specificity of Rossmax X5 in detecting atrial fibrillation

Statistic	Value	95% CI
Sensitivity	100.0%	93.02-100.00%
Specificity	98.6%	92.50-99.96%
Positive predictive value	98.1%	91.14-99.80%
Negative predictive value	100.0%	93.02-100.00%
Disease prevalence	50.0%	
Accuracy	99.3%	95.76-99.99%

## Discussion

This study was conducted among patients from the INR clinic and the cardiology clinic. These clinics are mainly selected because they have a greater number of patients with AF, as well as among the concentrated number of patients with all risk factors for AF. The purpose is to identify the accuracy of Rossmax X5, an ABPM, to detect AF among these patients, and, eventually, this device can be suggested to be used as a screening tool for AF detection despite its usual usage in the clinic as a blood pressure measurement tool. This is as suggested by guidance from the National Institute for Health and Care Excellence (NICE) which advocates the use of automated BPMs for the detection of suspected AF in patients being screened or monitored for hypertension [10]. Furthermore, we use ABPM because it is readily available and cost-effective as it is affordable as compared to an ECG machine.

Rossmax X5 is designed to detect abnormal rhythm, including AF despite its routine use as a BPM. It is integrated with pulse arrhythmia (PARR) technology that enables to detect rhythm abnormalities, namely premature contraction and AF. In this study, we just concentrate on the accuracy of AF detection because AF is a well-known condition associated with more severe complications yet preventable, namely stroke.

The accuracy of any device can be determined by measuring the sensitivity, specificity, and positive and negative predictive values. Sensitivity is the probability that a test gives a positive diagnosis, given that the individual actually has the condition for which the person is being tested. In other words, it is the accuracy of the screening test in identifying disease in people who genuinely have the disease, whereas specificity is the probability that a test yields a negative diagnosis given that the individual does not have the condition for which the person is being tested. If the result of a highly specific test for the individual is positive, the individual will be most likely to have the disease. These parameters are usually influenced by two factors, which are the spectrum of the disease and the patient characteristics. However, they are not affected by disease prevalence.

In this study, we found out that Rossmax X5 is very excellent in the detection of AF with a sensitivity of up to 100% and a specificity of 98%. This contributes to its accuracy of 99% for AF detection. Among patients with AF, Rossmax X5 can detect all of them. This study is comparable to the research that has been performed for other BPMs, Microlife and OMRON devices, which showed a sensitivity of around 98% and specificity of 97% [15]. Apart from that, there was only one subject with sinus pause that was falsely detected as AF. This probably may be due to the irregularity of the rhythm that leads to identifying the rhythm as AF falsely.

The sensitivity and specificity are essential measures of the accuracy of a test but cannot be used to estimate the probability of the disease in an individual patient. This can be provided by positive and negative predictive values, but both parameters vary according to disease prevalence. Positive predictive value is the probability of subjects with a positive screening test truly having the disease. Negative predictive value is the probability of subjects with a negative screening test truly not having the condition [16]. In a nutshell, screening in a high-risk group is more effective.

We found that Rossmax X5 has a positive predictive value of 98.6% and a negative predictive value of 100%. These values reflect that Rossmax X5 is a rather excellent device as a screening tool.

The traditional way for detection of AF is by manual pulse checking for irregularities and requires confirmation by a 12-lead ECG [17]. Currently, in our clinic, we are using ABPMs without integrated technology to detect AF. Owing to the high workload in the clinic, we usually tend to miss pulse-check during blood pressure measurement. So, by using ABPMs integrated with AF detection technology, we can reduce the opportunity to miss patients with asymptomatic AF. This was projected by the previous study by Taggar et al., which found that BPMs have substantially greater accuracy for detecting pulse irregularities caused by AF than pulse palpation [18]; such devices are likely to be practical alternatives to pulse palpation as blood pressure checks are an integral component of existing cardiovascular examination.

Furthermore, automated devices would enable screening to be conducted by all healthcare professionals without the need for additional training [19]. However, to date, there have been no economic analyses comparing ABPMs to pulse palpation for detecting suspected AF. This would help to inform optimal planning and service configurations of any future AF screening program [20,21]. Besides, the ABPM is easily, widely available and cost-effective compared to an ECG machine.

Paroxysmal AF is a condition that occurs when the episode of irregular rhythm terminates spontaneously or with intervention in less than seven days. Few patients have underlying AF but not detected during the procedures. The limitation of the ABPM is that it cannot detect AF in this group of people because of the episodic nature of the AF. They need to undergo an ECG recording of 24 hours or more such as Holter in order to detect AF. 

Study limitation

This study is not without limitations. First, cases were known to the investigator before the test. This leads to selection bias, and this can be minimized by the randomized selection of patients. Secondly, screening using ABPM may not be able to detect paroxysmal AF, owing to the episodic nature of AF; therefore, continuous ECG recording such as Holter is the best approach to identify these patients and should be used in a specific population [22]. Our study only took patients in the INR and cardiology clinics with a high prevalence of AF; therefore, it would have affected its applicability to the general population. Finally, the ABPM cannot detect any other arrhythmias apart from AF. Thus, the result from this study is only valid for AF patients.

Recommendation

There are a few recommendations that can be identified to improve future studies. Screening of AF using ABPM should be performed not only in the medical specialist clinic but also in other clinics, especially in the primary care setting. This is because there are many patients with risk factors of AF who come for regular follow-up in this clinic. Furthermore, this study may be confounded by selective bias. Blinding the investigator is the suggested way to reduce the possibility of bias. Apart from that, more participants with cardiac rhythm abnormality other than AF should be recruited in future studies so that we can assess more about the accuracy of Rossmax X5 to detect them.

## Conclusions

This study has shown that Rossmax X5 has successfully detected the high prevalence of AF patients with good accuracy. Hence, this kind of specialized BPM can be recommended as an alternative to conventional BPMs in daily clinical practice, especially among patients with a high risk of AF. This is owing to the attributed complication of AF such as stroke that can lead to an increase in morbidity and mortality and a burden to the socioeconomic condition of a society.
